# Association of serum inflammatory markers in early-pregnancy with the risk for gestational diabetes mellitus: a prospective cohort in Shenzhen, China

**DOI:** 10.3389/fendo.2025.1486848

**Published:** 2025-02-27

**Authors:** Yijin Wang, Qinqin Ren, Hui Yuan, Yang Wang, Yao Liu, Yuanhuan Wei, Ruifang Sun, Hongguang Yang, Ping Tian, Jianjun Yang, Guifang Deng

**Affiliations:** ^1^ Department of Public Health, Ningxia Medical University, Yinchuan, Ningxia, China; ^2^ Department of Clinical Nutrition, Shenzhen Nanshan People’s Hospital, Shenzhen, Guangdong, China; ^3^ Department of Children Healthcare, Shenzhen Nanshan People’s Hospital, Shenzhen, Guangdong, China

**Keywords:** systemic immune-inflammation index, systemic inflammation response index, inflammation, gestational diabetes mellitus, pregnant, first-trimester, prospective cohort study

## Abstract

**Introduction:**

The systemic immune-inflammation index (SII) and systemic inflammation response index (SIRI) have recently been reported as novel inflammatory markers of diabetes. However, the associations of SII and SIRI with the risk of gestational diabetes mellitus (GDM) are unclear. In our study, we explored the association between the SII and SIRI in early pregnancy and the risk of GDM in pregnant women.

**Methods:**

A prospective cohort of 1,505 pregnant women were recruited at 6–13 weeks of gestation in 2019 and 2020 in Shenzhen, China. SII and SIRI were determined by calculating the composite inflammation indicators from routine blood test results at 6–13 weeks of gestation, and an oral glucose tolerance test was conducted at 24–28 weeks of gestation to diagnose GDM. Logistic regression was used to analyse the correlations between the incidence of GDM and SII and SIRI. Using a restriction cubic spline with baseline SII and SIRI as continuous variables, the dose–response associations between the incidence of GDM and SII and SIRI were explored.

**Results:**

Following Ln-transformation of the SII and SIRI, multivariate models showed that Ln (SII) (odds ratio [OR] = 1.759; 95% confidence interval [CI]: 1.272–2.432) and Ln (SIRI) (OR = 1.556; 95% CI: 1.187–2.042) were positively associated with the risk of GDM in a dose-dependent manner. The OR for the highest quartile of SII compared with the lowest quartile for the risk of GDM was 2.080 (95% CI: 1.447–2.990), and the OR for the highest quartile of SIRI compared with the lowest quartile was 1.694 (95% CI: 1.170–2.452). The restricted cubic spline model confirmed a linear association between Ln (SII) and Ln (SIRI) with the risk of GDM (p-nonlinear > 0.05).

**Discussion:**

Higher SII and SIRI in early pregnancy are associated with an increased risk of GDM. As novel, valuable, and convenient indicators of inflammation, SII and SIRI could be used to a potential predictor for GDM in early pregnancy.

## 1 Introduction

As a common complication of pregnancy, GDM is characterised by an inadequate pancreatic response to insulin (1). GDM affects 14% of pregnancies globally (2), and the prevalence of GDM in China is 14.8% (3). GDM poses immediate and long-term health risks for both mother and child, such as pre-eclampsia, premature membrane rupture, shoulder dystocia, macrosomia, perinatal death, and future risks of hyperglycemia, diabetes, obesity, and cardiovascular disease (4–6). Adverse metabolic programmes in the offspring may exist prior to the diagnosis of GDM (7, 8). Therefore, there is a need for more effective indicators to predict GDM and control the risk factors.

Tumor necrosis factor (TNF) levels in pregnant women have been reported to be significantly and negatively correlated with insulin sensitivity (9, 10). Several inflammatory markers, such as interleukins, C-reactive protein, tumour necrosis factor-α, leukocytes, neutrophils, and monocytes, were associated with GDM (11–13). Considering the inherent susceptibility of inflammatory parameters to other factors, the neutrophil-to-lymphocyte ratio (NLR), platelet-to-neutrophil ratio, platelet-to-lymphocyte ratio (PLR), and monocyte-to-lymphocyte ratio (MLR) could be used in combination as theoretically more reliable markers for assessing inflammatory status than individual cells. Wenhua Liu et al. found that increased PLR and NLR were independent predictors of GDM (14). A systematic review and meta-analysis also reported higher NLR in GDM pregnancies than in normoglycemic controls recently (15). However, the progression of gestational diabetes is a complex biological process, and there is limitation to analyze the relationship between single biomarkers and the risk of GDM. Therefore, integrating more biomarker data for analysis could make up for the data problems caused by data loss, noise and other factors in single data analysis, and reduce the false positive caused by single biomarker analysis.

The Systemic Immune-Inflammation Index (SII) and the Systemic Inflammation Response Index (SIRI) are emerging biomarkers that are derived from the counts of platelets, neutrophils, lymphocytes, and monocytes. These indices have been validated for their ability to offer a comprehensive assessment of an individual’s inflammatory and immune status (16, 17). The SII and SIRI have been found to more reliably predict disease progression and prognosis in certain inflammation-related diseases, such as cardiovascular disease (18, 19) and cancer (20, 21). Recently SII and SIRI as straightforward and cost-effective indexs identifying individuals with insulin resistance, diabetes and its complications have been reported (22, 23). However, few studies focused on the association between SII and SIRI and GDM, and those that have been published have reported ambiguous conclusions. Only a few retrospective or observational studies reported higher SIRI and SII in the GDM group than the non-GDM group (24–26) from which causal inference could not be performed.

In this study, a prospective cohort was designed to explore the association of serum inflammatory markers in early-pregnancy with the risk for gestational diabetes mellitus in Shenzhen city, in which migrants account for more than 70% of urban residents, ranking first among the top ten immigrant cities in China.

## 2 Materials and methods

### 2.1 Study population

Our study was conducted from 2019 to 2020 at Shenzhen Nanshan People’s Hospital, Shenzhen, China. Pregnant women were followed from their first antenatal visit until delivery. A cohort of pregnant women was established based on the following inclusion criteria: 1) age ≥ 18 years; 2) singleton pregnancy; and 3) availability of complete medical records. Potential participants were excluded if they had: 1) twin or multiple pregnancies (n = 9); 2) pre-conceptional diabetes (n = 6); 3) hepatitis or impaired liver function (n = 74); 4) nephritis or impaired renal function (n = 9); 5) cardiovascular disease (n = 3); 6) autoimmune disease (n = 1); or 7) an acute or chronic inflammatory condition during pregnancy or an infectious disease (n = 73). We restricted the study sample to pregnant women who underwent routine blood measurements from 6 to 13 weeks of gestation and three glucose measurements using the oral glucose tolerance test (OGTT), resulting in 1,505 pregnant women included in the analysis. Clinical information was obtained from the hospital record information system and was analysed anonymously. Baseline characteristics were obtained either through a standardised self-report questionnaire or a face-to-face interview. Ethical approval for this cohort was granted by the Ethics Committee of Shenzhen Nanshan People’s Hospital. All subjects provided informed consent. The study was conducted in accordance with the guidelines of the Declaration of Helsinki, as set forth by the World Medical Association.

### 2.2 Data collection and clinical measurements

A structured questionnaire and review of the hospital information system records were used to collect information on maternal age, weight, height, educational level, smoking status, alcohol consumption, family history, and pregnancy history. Maternal body mass index (BMI, kg/m^2^) was calculated as the weight (kg) divided by the square of the height (m^2^). A BMI > 24 kg/m^2^ was defined as overweight. Smokers were defined as those who smoked one or more cigarettes per day, and alcohol consumption was defined as drinking at least one alcoholic beverage per week. Educational attainment was classified as primary, secondary, or college and above. Parity was defined as the number of live or stillborn infants prior to the current pregnancy.

At 6–13 weeks of gestation, peripheral venous blood was drawn for biochemical index testing. The participants were asked to fast the night before the blood was drawn. All samples were immediately centrifuged after standing for laboratory measurements. The neutrophil count, lymphocyte count, platelet count, white blood cell count, and monocyte count were measured using a haematology analyser (Sysmex XN90000; Sysmex, Kobe, Japan). SII and SIRI are derived from blood cell counts in samples that have been stringently tested using standardized protocols. These indices offer valuable insights into systemic inflammation and immune status.

### 2.3 SII/SIRI and covariates

SII was calculated using the formula (platelet count (×10^9^/L)× neutrophil count (×10^9^/L))/lymphocyte count (×10^9^/L), and SIRI was calculated using the formula (monocyte count (×10^9^/L) × neutrophil count (×10^9^/L)/lymphocyte count (×10^9^/L). The analyses included potential confounding factors associated with the SII, SIRI, and GDM, based on previous studies. The covariates were age, BMI, educational level, smoking status, alcohol consumption, family history of hypertension, family history of diabetes mellitus, number of pregnancies, parity, and history of miscarriage.

### 2.4 Assessment of GDM

Participants underwent a 75 g oral glucose tolerance test (OGTT) during the 24-28th week of pregnancy. 75 g of glucose was given and maternal blood samples were taken at 0, 1, and 2 h to measure serum glucose concentration. According to the recommendations of the International Association of the Diabetes and Pregnancy Study Groups Consensus Panel, all pregnant women receiving a 75-g OGTT at 24–28 weeks of gestation were diagnosed with GDM when one of the following criteria was met: fasting plasma glucose concentration ≥ 5.1 mmol/L; 1-h plasma glucose concentration ≥ 10.0 mmol/L; or 2-h plasma glucose concentration ≥ 8.5 mmol/L.

### 2.5 Statistical analysis

The Kolmogorov-Smirnov test was used to determine whether continuous variables conformed to normal distribution. The independent samples t-test was used for normally distributed variables and the Mann-Whitney U-test was used for non-normally distributed variables. And the chi-square test was used for categorical variables. To investigate the association of SII and SIRI with the risk of GDM, we performed a multivariate logistic regression analysis. Model 1 had no covariates; Model 2 included age and BMI as covariates; and Model 3 included age, BMI, educational level, smoking status, alcohol consumption, family history of diabetes mellitus, family history of hypertension, number of pregnancies, parity, and history of miscarriages. The associations of SII and SIRI with the risk of GDM were assessed using odds ratios (ORs) and 95% confidence intervals (CIs). The dose–response association between GDM and SII was explored using restricted cubic spline (RCS) curves adjusted for potential confounders, with the SII as the continuous variable. Subgroup analyses were performed according to age and BMI. Data were analysed using R software version 3.0.3 (The R Foundation for Statistical Computing, Vienna, Austria) and SPSS 24.0 software (SPSS Inc., Chicago, IL, USA). A two-tailed *p*-value < 0.05 was defined as statistically significant.

## 3 Results

### 3.1 Baseline characteristics

We conducted a prospective study of 1,505 pregnant Chinese women (**Table 1**). Compared with those without GDM, those with GDM were older, had a higher BMI, and had a greater frequency of multiparity (*p* < 0.001). Those with GDM also had higher fasting plasma glucose, 1-hour post-load glucose, and 2-hour post-load glucose concentrations and higher blood pressure (*p* < 0.05). Furthermore, the patients with GDM had higher platelet counts, neutrophil counts, white blood cell counts, SII and SIRI than those without GDM (*p* < 0.05). No significant differences between groups were observed for the other indicators (all *p* > 0.05).

**Table 1:** Baseline characteristics.

Variable	GDM	Non-GDM	*P* value
N	337	1168	
Age	35.56 ± 4.36	33.62 ± 3.93	**<0.001**
Age categories			**<0.001**
< 35	148 (43.9%)	738 (63.2%)	
≥ 35	189 (56.1%)	430 (36.8%)	
BMI (kg/m^2^)	21.51 ± 2.84	20.44 ± 2.50	**<0.001**
BMI categories			**<0.001**
<18.5	41 (12.2%)	259 (22.2%)	
18.5-24	234 (69.4%)	806 (69%)	
>24	62 (18.4%)	103 (8.8%)	
Education			0.994
Primary	30 (8.90%)	105 (9.0%)	
Secondary	55 (16.3%)	188 (16.1%)	
College or above	252 (74.8%)	875 (74.9%)	
Smoking status (%)	1 (0.3%)	2 (0.2%)	0.649
Alcohol status (%)	1 (0.3%)	1 (0.1%)	0.349
Diabetes history (%)	10 (3%)	23 (2%)	0.27
Hypertension history (%)	16 (4.7%)	39 (3.3%)	0.225
Conception method			0.212
Natural	328 (97.3%)	1149 (98.4%)	
Artificial	9 (2.7%)	19 (1.6%)	
Parity			**<0.001**
Primiparity	170 (50.4%)	717 (61.4%)	
Multiparity	167 (49.6%)	451 (38.6%)	
History of miscarriage	103 (30.6%)	301 (25.8%)	0.08
OGTT			
Fasting plasma glucose (mmol/L)	4.76 ± 0.40	4.49 ± 0.28	**<0.001**
1-h post-load glucose (mmol/L)	9.65 ± 1.44	7.51 ± 1.28	**<0.001**
2-h post-load glucose (mmol/L)	8.67 ± 1.34	6.51 ± 1.00	**<0.001**
Blood pressure (mmHg)			
SBP (mm Hg)	112.23 ± 12.03	109.75 ± 12.23	**<0.001**
DBP (mm Hg)	68.09 ± 8.32	66.47 ± 8.21	**0.002**
WBC (x10^9^/L)	8.80 ± 1.93	8.33 ± 1.94	**<0.001**
Platelet (x10^9^/L)	250.86 ± 52.21	243.10 ± 50.99	**0.007**
Neutrophil (x10^9^/L)	6.32 ± 1.68	5.90 ± 1.66	**<0.001**
Lymphocyte (x10^9^/L)	1.83 ± 0.50	1.79 ± 0.46	0.155
Monocyte (x10^9^/L)	0.53 ± 0.14	0.51 ± 0.16	0.054
SII(x10^9^/L)	913.7 ± 365.38	841.9 ± 342.45	**<0.001**
SIRI(x10^9^/L)	1.75 ± 0.96	1.62 ± 0.92	**0.003**

Data was presented as Mean (± SD) for continuous variables and n (%) for categorical. BMI, body mass index; OGTT, oral glucose tolerance test; SBP, systolic blood pressure; DBP, diastolic blood pressure; WBC, white blood cell count; SII, systemic immune-inflammation index; SIRI, systemic inflammation response index. Bold font indicates P<0.05.

### 3.2 Association between GDM and SII

**Figure 1:**
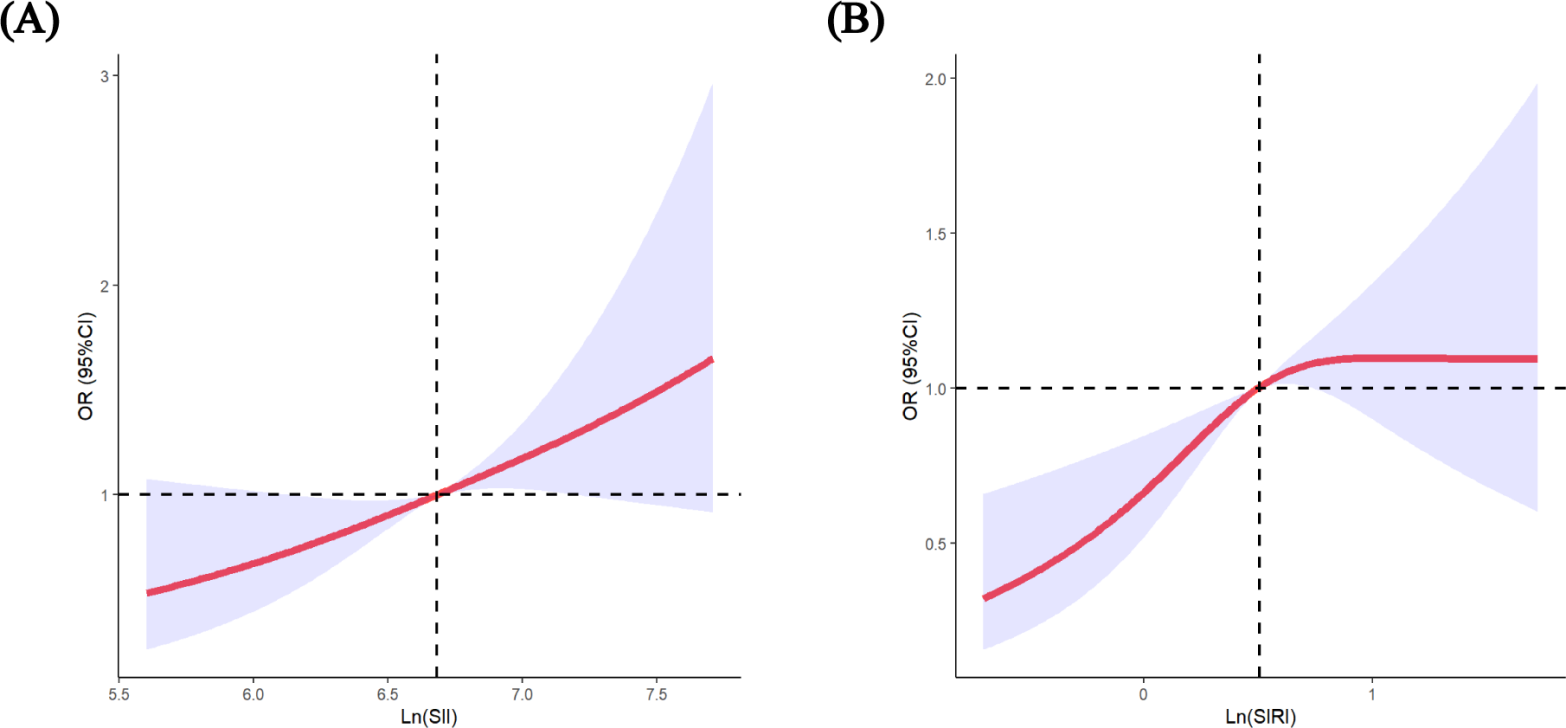
Restricted cubic spline (RCS) regression analysis including **(A)** SII (p-nonlinear=0.147) **(B)** SIRI (p-nonlinear=0.015) with GDM risk. The median of SII 800.81, SIRI 1.65 was selected as the reference levels, respectively. The lines indicate estimated ORs, and the light-blue–shaded areas represent 95% CI.

Following established methods, we logarithmically transformed SII and SIRI to normalize distributions and better detect associations with GDM risk (27–29). As presented in **Table 2**, the Ln (SII) was positively associated with GDM in the crude model and in Model 2 (adjusted for age and BMI). In Model 3 (adjusted for all variables), this positive association remained stable (OR = 1.759; 95% CI: 1.272-2.432; p = 0.001). Similar results were obtained when the data were analysed according to SII quartiles. After adjustment for multiple variables, the OR for the highest quartile of the SII compared with the lowest quartile was 2.080 (95% CI: 1.447–2.990). Moreover, the *p*-values for the trend of the three models were all less than 0.05. Further analysis using RCS confirmed a linear association between the SII and the risk of GDM (*p* for non-linearity = 0.147, **Figure 1**). The RCS regression model revealed that higher SII were associated with an increased risk of GDM.

**Table 2:** ORs (95%CI) for GDM according to the quartiles of SII.

Variables	Model1		Model2		Model3	
	OR (95%CI)	*P*-Value	OR (95%CI)	*P*-Value	OR (95%CI)	*P*-Value
Ln (SII)	1.753 (1.278-2.404)	**<0.001**	1.728 (1.252-2.383)	**0.001**	1.759 (1.272-2.432)	**0.001**
SII quartiles						
Quartiles 1	Reference		Reference		Reference	
Quartiles 2	1.412 (0.981-2.032)	0.063	1.481 (1.020-2.151)	**0.039**	1.499 (1.029-2.184)	**0.035**
Quartiles 3	1.412 (0.981-2.032)	0.063	1.341 (0.925-1.946)	0.122	1.332 (0.914-1.940)	0.135
Quartiles 4	2.009 (1.414-2.853)	**<0.001**	2.035 (1.420-2.916)	**<0.001**	2.080 (1.447-2.990)	**<0.001**
*P* for trend		**<0.001**		**<0.001**		**<0.001**

Model 1 had no covariates, Model 2 added covariates age and BMI, and Model 3 adjusted variables: age, BMI, education, smoking status, alcohol consumption, family history of diabetes mellitus, family history of hypertension, number of pregnancies, parity, and history of miscarriages. 95%CI, 95% confidence interval; OR, odds ratio; SII, systemic immune-inflammation index. a p < 0.05 was considered statistically significant. Bold font indicates P<0.05.

**Figure 2:**
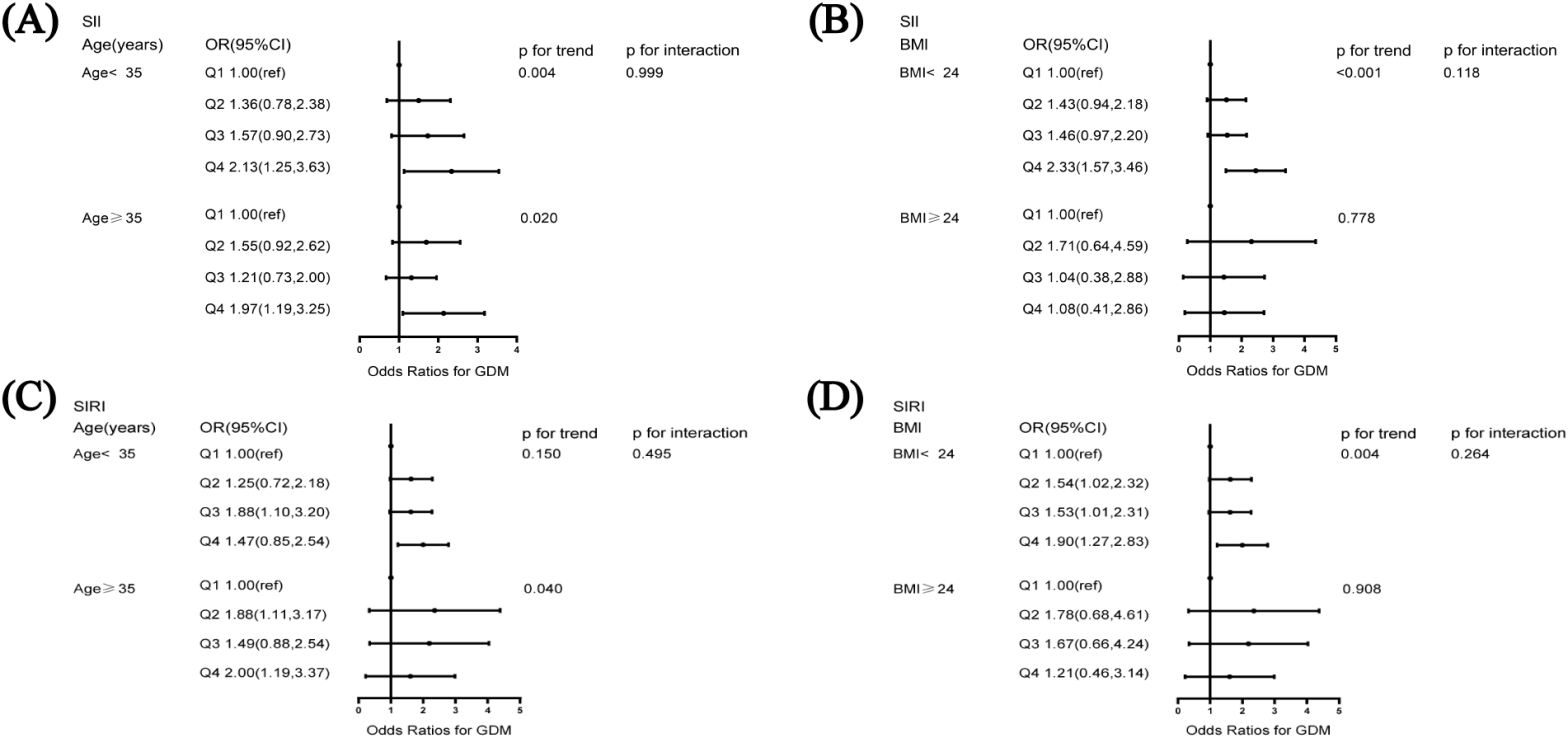
Subgroup analysis of the association of systemic immune-inflammation index and systemic inflammation response index with GDM **(A)** association of SII and GDM stratified by age, **(B)** association of SII and GDM stratified by BMI, **(C)** association of SIRI and GDM stratified by age, **(D)** association of SIRI and GDM stratified by BMI. The model was adjusted for age, BMI, education, smoking status, alcohol consumption, family history of diabetes mellitus, family history of hypertension, number of pregnancies, parity, and history of miscarriage. BMI, body mass index.

Analyses stratified by age showed that, among participants aged < 35 years, the risk of GDM was 2.13-fold higher among those in the highest quartile of SII than among those in the lowest quartile (95% CI: 1.25–3.63). Moreover, among participants aged ≥ 35 years, the OR for the highest quartile of SII as compared with the lowest quartile was 1.97 (95% CI: 1.19–3.25). A similar analysis for the subgroup of women with a pre-pregnancy BMI < 24 kg/m^2^ yielded an OR of 2.33 (95% CI: 1.57-3.46). We did not find significant interactions between the SII, age, or BMI and GDM (*p* for interaction > 0.05, **Figure 2**).

### 3.3 Relationship between GDM and SIRI

The association between SIRI and the risk of developing GDM is depicted in **Table 3**. A positive association between the Ln (SIRI) and the risk of GDM was observed in the crude model. After adjusting for confounding factors, these associations were largely similar to those observed in the crude models (OR = 1.556; 95% CI: 1.187-2.042; p = 0.001). Sensitivity analyses using the SIRI quartile as a categorical variable were consistent with the results of the main analysis. In Model 3, the ORs (95% CI) were 1.543 (1.064–2.239), 1.606 (1.104–2.335), and 1.694 (1.170–2.452) for SIRI in quartiles 2, 3 and 4, respectively, compared with quartile 1. In addition, the *p*-values for the trends in GDM risk were significant in all models. There was a significant linear association between the Ln(SIRI) and the risk of GDM (p for non-linearity>0.05, **Figure 1**).

**Table 3:** ORs (95%CI) for GDM according to the quartiles of SIRI.

Variables	Model1		Model2		Model3	
	OR (95%CI)	*P*-Value	OR (95%CI)	*P*-Value	OR (95%CI)	*P*-Value
Ln (SIRI)	1.520 (1.168-1.976)	**0.002**	1.530 (1.170-2.001)	**0.002**	1.556 (1.187-2.042)	**0.001**
SIRI quartiles						
Quartiles 1	Reference		Reference		Reference	
Quartiles 2	1.568 (1.095-2.247)	**0.014**	1.511 (1.046-2.182)	**0.028**	1.543 (1.064-2.239)	**0.022**
Quartiles 3	1.523 (1.062-2.185)	**0.022**	1.597 (1.104-2.311)	**0.013**	1.606 (1.104-2.335)	**0.013**
Quartiles 4	1.709 (1.197-2.44)	**0.003**	1.654 (1.148-2.383)	**0.007**	1.694 (1.170-2.452)	**0.005**
*P* for trend		**0.011**		**0.017**		**0.015**

Model 1 had no covariates, Model 2 added covariates age and BMI, and Model 3 adjusted variables: age, BMI, education, smoking status, alcohol consumption, family history of diabetes mellitus, family history of hypertension, number of pregnancies, parity, and history of miscarriages. 95%CI, 95% confidence interval; OR, odds ratio; SIRI, systemic inflammation response index. a p < 0.05 was considered statistically. Bold font indicates P<0.05.

For participants aged ≥ 35 years, the risk of developing GDM was 2.00 times higher (95% CI: 1.19–3.37) for those in the top quartile of SIRI than for those in the bottom quartile. Moreover, among participants with a BMI < 24 kg/m^2^, the OR for the highest quartile of SIRI compared with the lowest quartile was 1.90 (95% CI: 1.27–2.83). **Figure 2** shows that neither age nor BMI significantly modified this positive correlation (*p* for interaction > 0.05).

## 4 Discussion

To the best of our knowledge, this is the first study to assess the association between SII and SIRI and the risk of GDM prospectively in a large cohort. We found that among pregnant women, a high SII or SIRI was strongly associated with an increased risk of GDM. This association remained significant after adjusting for confounders. In conclusion, SII and SIRI can be used as effective and relatively inexpensive predictors for assessing the risk of GDM in pregnant women.

Recently, there has been increasing evidence that the development of GDM may be associated with a low-grade systemic inflammatory state and placental inflammation during pregnancy (30–32). The inhibition of insulin secretion occurs due to the secretion of pro-inflammatory cytokines and the mediation of pro-inflammatory signalling pathways (33). For example, pro-inflammatory cytokines, such as TNF - α, cause insulin resistance by promoting adipocyte catabolism and increasing serine/threonine phosphorylation of insulin receptor substrate-1 (IRS-1) through a variety of signalling pathways, including the IKKβ/NF-κB pathway (34). Some immune-related genes have been found to be highly expressed in the placentas of patients with GDM, and the proportion of M1 macrophages is higher in these placentas than in control placentas (35). Increased M1 macrophages secrete pro-inflammatory mediators such as TNF-α, IL-1β and resistin, which act on adipocytes to induce a state of insulin resistance, leading to a positive feedback loop of inflammation and insulin resistance (36). In addition, excess adipose tissue enhances circulating monocyte recruitment and increases the production of pro-inflammatory cytokines (37). Inflammatory cytokines stimulate an increase in insulin secretion, and excess insulin secretion further impairs pancreatic islet function, leading to glucose metabolism disorders (38).

There are a few studies reported inflammatory markers as predictor for the risk of diabetes (39, 40). However limited evidence is reported to explore the association between inflammation and GDM in retrospective and cross-sectional studies. Yildiz et al. found that SIRI and SII in the first trimester were significantly higher in the GDM group than in the non-GDM group in a prospective observational study of 201 pregnancies (24). In a retrospective study of 467 pregnancies, Ergani et al. found that SII were higher in the GDM group than the non-GDM group in the late stages of pregnancy. Moreover, SII significantly increased from early to late pregnancy in patients with GDM (25). However, causal inference could not be performed from these retrospective and cross-sectional studies, Moreover, the effects of confounding factors were not evaluated and subgroup analyses were not conducted. Xiaoyan Xiu et al. found in a retrospective study that higher levels of SII and SIRI were associated with pregnancy outcomes in patients with GDM (26). However, the relationship between inflammation index and gestational diabetes mellitus was not investigated. Our study used a different study design, sample size, biomarker assessment time, diagnostic criteria or grading level compared to previous studies. We extended these findings to a relatively large prospective cohort study of pregnant Chinese women and observed that those in the second quartile of SII had a 49.9% higher risk of developing GDM in early pregnancy, while those in the fourth quartile had a 2.08 times higher the risk of developing GDM in early pregnancy, compared with those in the first quartile. We also found that pregnant women with SIRI in the second, third, and fourth quartile had a 54.3%, 60.6%, and 69.4% higher risk, respectively, of GDM in early pregnancy than those with SIRI in the first quartile. To the best of our knowledge, this is the first prospective large cohort study to assess the association between SII and SIRI and the risk of GDM.

According to Huang et al., platelet counts were significantly higher in the GDM group than the non-GDM group (41). Moreover, Ye et al. found that white blood cells, neutrophils, lymphocytes, monocytes, and the NLR in early pregnancy were positively associated with the risk of GDM (13). Similarly, we found that platelet counts, neutrophil counts, and white blood cell counts were higher in the GDM group than the non-GDM group. A meta-analysis found that the NLR was significantly elevated in patients with GDM but was not significantly correlated with the PLR, which is also a component of the SII (42). A case-control study suggested that an increased white blood cells, PLR, and NLR may be causally associated with an increased risk of GDM (14). The SII and SIRI selected for use in this study included the platelet count, neutrophil count, lymphocyte count, and monocyte count, while the PLR and NLR were components of the SII and SIRI, respectively. The experimental results truly reflected the burden of inflammation and accurately reflected the associations of SII and SIRI with GDM.

Excess adipose tissue has been found to lead to enhanced recruitment of circulating monocytes and increased production of pro-inflammatory cytokines, thus affecting insulin resistance (33). In addition, as humans age, the number and activity of cells change, and the body’s balance changes, increasing susceptibility to related diseases (43). Age and obesity are also strongly associated with GDM (44–46). Considering that both age and obesity affect the risk of GDM and inflammation, we attempted, for the first time, to analyse the association between SII and SIRI and the risk of GDM using subgroups according to age and BMI. Neither age nor BMI had a significant effect on the associations of SII and SIRI with GDM in subgroup analyses. This suggests that the associations between higher SII or SIRI and the risk of GDM remain reliable, regardless of age or BMI.

Currently, the SII and SIRI are used as reliable indicators of inflammatory status to predict certain diseases, such as cardiovascular disease (18, 19) and cancer (20, 21),. Routine blood tests are widely used in clinical practice. SII and SIRI can be obtained through such tests. Blood samples are easy to obtain, and the routine tests are simple to operate, minimally invasive, and inexpensive. SII and SIRI contain measurements for three inflammatory cell types, which may better characterise the complex inflammatory mechanisms associated with GDM than indices comprising single or dual inflammatory cell types. In clinical practice, the SII and SIRI are expected to serve as biomarkers for the early identification and risk assessment of GDM.

Our study has several strengths and limitations. First, to the best of our knowledge, this is the first prospective large-sample cohort study to assess the associations of SII and SIRI with GDM in a population of pregnant Chinese women. Second, our study included confounding factors in the analyses, as much as possible, to investigate the utility of the SII and SIRI for the prediction of GDM. SII and SIRI were transformed into categorical variables to obtain consistent results and improve the stability of the data. RCS was used to analyse possible nonlinear associations between SII and SIRI and the risk of GDM. In addition, stratified analyses were performed to assess the effects of SII and SIRI. The measurement of SII and SIRI will help to stratify the risk of GDM and provide personalised guidance for its clinical management. Notably, there are some limitations of this study. First, we adjusted for a number of potential covariates. However, we could not completely exclude the effects of other unmeasured or residual confounders. Second, we performed subgroup analyses based only on age and BMI, and these analyses had limited statistical power. Finally, all study participants were from Shenzhen, China, and this relatively limited population source may have introduced selection bias.

## 5 Conclusions

The present study suggests that higher SII and SIRI increase the risk of GDM, and that SII and SIRI may serve as a direct and economical tool for predicting GDM in early pregnancy. Further studies are needed to confirm our findings.

## Data Availability

The raw data supporting the conclusions of this article will be made available by the authors, without undue reservation.
